# Probiotic treatment with *Bifidobacterium animalis* subsp. *lactis* LKM512 + arginine improves cognitive flexibility in middle-aged mice

**DOI:** 10.1093/braincomms/fcad311

**Published:** 2023-11-13

**Authors:** Daisuke Joho, Masahira Takahashi, Takeru Suzuki, Kayo Ikuta, Mitsuharu Matsumoto, Masaki Kakeyama

**Affiliations:** Laboratory of Environmental Brain Science, Faculty of Human Sciences, Waseda University, Tokorozawa 3591192, Japan; Laboratory of Environmental Brain Science, Faculty of Human Sciences, Waseda University, Tokorozawa 3591192, Japan; Laboratory of Environmental Brain Science, Faculty of Human Sciences, Waseda University, Tokorozawa 3591192, Japan; Dairy of Science and Technology Institute, Kyodo Milk Industry Co, Ltd., Tokyo 1900182, Japan; Dairy of Science and Technology Institute, Kyodo Milk Industry Co, Ltd., Tokyo 1900182, Japan; Research Institute for Environmental Medical Sciences, Waseda University, Tokorozawa 3591192, Japan; Laboratory of Environmental Brain Science, Faculty of Human Sciences, Waseda University, Tokorozawa 3591192, Japan; Research Institute for Environmental Medical Sciences, Waseda University, Tokorozawa 3591192, Japan

**Keywords:** touchscreen, mouse, polyamines, microbiota–gut–brain axis, cognitive flexibility

## Abstract

Cognitive flexibility, the ability of adapting to an ever-changing environment, declines with aging and impaired in early stages of dementia. Although recent studies have indicated there is a relationship between the intestinal microbiota and cognitive function, few studies have shown relationships between intestinal microbiota and cognitive flexibility because of limited behavioural tasks in mice. We recently established a novel cognitive flexibility task for mice using a touchscreen operant apparatus and found that probiotic treatment with a mixture of *Bifidobacterium animalis* subsp. *lactis* LKM512 and arginine improved cognitive flexibility in young adult mice. To confirm the effects of the probiotic treatment on cognitive flexibility and to determine whether it is effective even in older age, we here examined the effects of long-term treatment with *Bifidobacterium animalis* subsp. *lactis* LKM512 and arginine on cognitive flexibility in middle-aged mice. From 8 to 15 months of age, mice received LKM + Arg or vehicle (controls) orally three times per week and were subjected to the cognitive flexibility task at 13–15 months old. In one of indices of cognitive flexibility, both *Bifidobacterium animalis* subsp. *lactis* LKM512 and arginine-treated mice and vehicle-treated mice showed progressively improved performance by repeating reversal tasks, with a small trend that *Bifidobacterium animalis* subsp. *lactis* LKM512 and arginine-treated mice showed better learning performance through reversal phases. With respect to the other index of cognitive flexibility, *Bifidobacterium animalis* subsp. *lactis* LKM512 and arginine-treated mice showed significantly fewer error choices than control mice at the reversal phase, i.e. *Bifidobacterium animalis* subsp. *lactis* LKM512 and arginine improved the performance of behavioural sequencing acquired in the previous phase, which allowed *Bifidobacterium animalis* subsp. *lactis* LKM512 and arginine-treated mice to show an early onset of shift to reversal contingency. Taken together, long-term treatment with *Bifidobacterium animalis* subsp. *lactis* LKM512 and arginine was found to improve cognitive flexibility in middle-aged mice, indicating that probiotic treatment might contribute to prevention of age-related cognitive decline.

## Teaser

Joho *et al.* reported that long-term treatment with *Bifidobacterium animalis* subsp. *lactis* LKM512 and arginine improved mouse cognitive flexibility in middle-aged adulthood (over 13 months). It is possible that improvement of brain–gut axis can improve cognitive decline with aging.

## Graphical Abstract

**Graphical Abstract:**
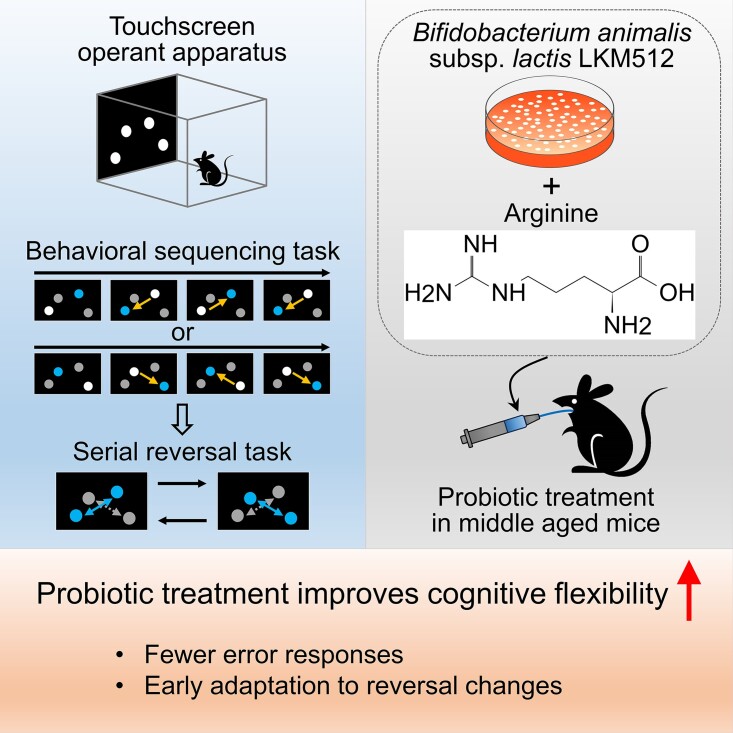

## Introduction

Cognitive flexibility, one of the executive functions, is the ability to adapt to a constantly changing environment.^1-5^ Cognitive flexibility declines with aging and is impaired by neurodegenerative diseases such as Alzheimer’s disease and mild cognitive impairment.^6,7^ The Wisconsin card sorting test and the Brixton spatial anticipation test are the major tools for assessing cognitive flexibility in humans.^8,9^ In mouse studies, cognitive flexibility has been assessed by measuring adaptation to a novel rule with changed spatial locations and visual discrimination contingencies.^10,11^ However, these protocols do not require subjects to utilize the prior knowledge acquired at a previous phase when facing a new situation. This would prevent a translational approach because humans ordinally use their prior knowledge even in response to a new situation.

We have previously established a task protocol to assess cognitive flexibility in mice using the IntelliCage system, a fully automated behavioural test apparatus in group-housed mice,^12^ and a touchscreen operant apparatus.^13,14^ Our cognitive flexibility task allows to assess differences of adaptation speed at the reversal phase and to measure cognitive flexibility in terms of mouse adaptation to contingency using prior knowledge.

The intestinal microbiota affects the bidirectional signalling pathway between the gut and the nervous system, in the so-called ‘microbiota–gut–brain axis’.^15^ For instance, differences in the composition of the intestinal microbiota in patients with mild cognitive impairment and dementia were observed,^16,17^ and cognitive impairment and lower expression of neural signalling-related molecules, including brain-derived neurotrophic factor in the medial prefrontal cortex, hippocampus, and hypothalamus, were observed in germ-free and antibiotic-treated mice.^18,19^ Moreover, the microbiota has been shown to influence not only cognitive function but also pathology and neuroinflammation in mouse models of Alzheimer’s disease.^20,21^ On the other hand, probiotic treatment improves cognitive function in patients with mild cognitive impaiment.^22^ Additionally, we recently reported that dysbiosis induces a decline in cognitive flexibility.^14^ The above evidence strongly supports a relationship between the intestinal microbiota and cognitive function.

A dietary intervention with functional food can be one of the tools for delaying and preventing cognitive decline during aging. With respect to the probiotic *Bifidobacterium animalis* subsp. *lactis* LKM512 (LKM) and arginine (Arg), we previously reported that LKM treatment in mice increased intestinal bacteria-derived polyamines, suppressed inflammation and promoted longevity^23^; furthermore, a combination of LKM and Arg enhanced these effects and improved spatial memory^24^ and cognitive flexibility in adulthood.^13^ Because cognitive flexibility is an important index of cognitive decline during aging and early onset of dementia, it is also necessary to carefully examine whether the effect of probiotic treatment on cognitive flexibility is even maintained in order age. Herein, we examined the effects of long-term treatment with LKM + Arg on cognitive flexibility in middle-aged mice.

## Materials and methods

### Animals

All animal experiments were reviewed by the Waseda University Review Committee for Animal Experiment and approved by the head of the institute (# A22–124). All animal experiments were conducted in accordance with the ethical guidelines of the Waseda University Review Committee for Animal Experiment. Male C57BL/6 mice (10 weeks old) were purchased from Oriental Yeast Co., Tokyo, Japan. All mice were maintained at the Animal Facility of Waseda University in accordance with the recommendation of the Standards relating to the Care and Keeping and Reducing Pain of Laboratory Animals, the Ministry of the Environment, Japan [22–24°C, with 40–60% humidity, under a 12/12 h light/dark cycle (lights on: 8:00 AM)]. All mice received food (MF, Oriental Yeast Co., Tokyo, Japan) and water *ad libitum* until the beginning of the study.

### Probiotic treatment

Twenty-four mice were randomly divided into two groups (LKM + Arg: *n* = 12, control: *n* = 12). All mice were received a Dulbecco’s phosphate-buffered saline (D-PBS) solution containing *B. animalis* subsp. *lactis* LKM512 and L-Arg mixture (LKM + Arg) or nothing (control). LKM512 was provided by Kyodo Milk Industry Co, Ltd. LKM512 was anaerobically preincubated at 37°C for 48 h by Anaero Pack Kenki (Mitsubishi Gas Chemical Company, Inc., Tokyo, Japan) on de Man Rogosa Sharpe agar medium. The colonies were suspended to absorbance 1.8–2.0/0.2 mL per mouse, and L-Arg was added to a concentration of 0.3 mg/g body weight. The solution was administered after the task three times per week via oral gavage using a sonde to control for an identical dosage, from 8 months of age. After 5 months of treatment (at 13 months of age), the cognitive flexibility task described below was conducted. The treatment continued during the task period.

### Touchscreen operant apparatus

**Figure 1:**
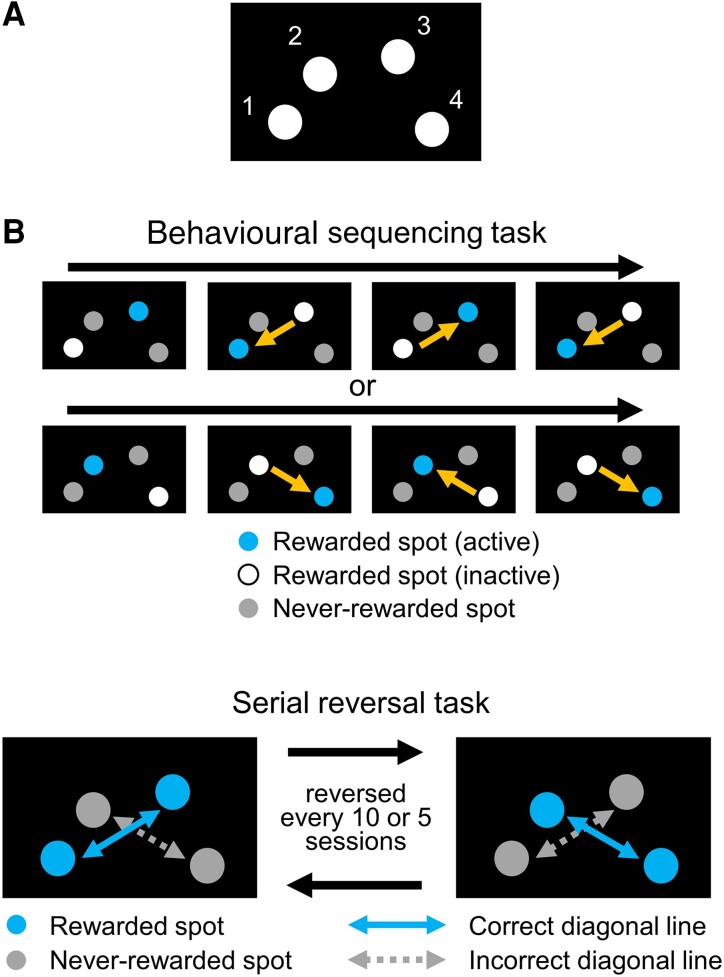
**Behavioural experiment to assess cognitive flexibility using a touchscreen operant apparatus.** (**A**) Position of spots. Mice are to choose one of four spots by nose poking into a hole positioned in front of a spot. All spots were lighting white during selection trials. (**B**) Diagram of behavioural sequencing task. Mice obtained a reward only by alternately choosing an active rewarded spot. When mice choose an active rewarded spot, the spot became an ‘inactive rewarded spot’. Choosing a never-rewarded spot was counted as an ‘error’. Mice had to learn a sequence of shuttling behaviours between two rewarded spots positioned diagonally. (**C**) Serial reversal learning of the behavioural sequencing task. The assignation of the two rewarded and never-rewarded spots was changed every 5 or 10 sessions. Alternative selection between the rewarded spots on the correct diagonal line was counted as a ‘diagonal correct’, and the alternative selection between the never-rewarded spots on the incorrect diagonal line was counted as a ‘diagonal error’.

The experiments were conducted using a touchscreen operant apparatus for mice (O’Hara & Co., Ltd., Tokyo, Japan). The apparatus was composed of a monitor, a touchscreen, a reward dispenser, a speaker, a house lamp, a camera and a chamber. Four spots were asymmetrically positioned on the screen (Fig. 1A). The mice could receive a 10 mg food pellet as a reward when they successfully chose a spot by nose poking into the hole on the screen. The operant system was automatically preceded by computer.

### Behavioural test procedure

The test procedure was mimicked based on our previously reported protocol.^13,14^ Mice were bred on food pellets [AIN-76A Rodent Tablet #1811213 (5TUL), TestDiet, St. Louis, MO, USA] as a reward during the task period. The mice were individually housed in their respective home cages, and dietary restriction continued to maintain the mouse body weight at ∼85% of that prerestriction to maintain mouse’s motivation for the rewards. All sessions were performed once a day.

### Acclimation

During the habituation phase, the mice were introduced into the chamber and explored freely. The mice received five reward pellets placed on the reward dispenser for the first half of 15 min; then, for the second half of the 15 min, the reward dispenser delivered one reward pellet every 30 s accompanied by a sound. This phase lasted 30 min per session and was conducted for two sessions. Thereafter, pretest shaping was conducted to train the mice to choose a spot to receive a reward by nose poking. First, the mice received a reward pellet accompanied by a sound if they chose illuminating the highest spot on the screen (Spot 3, Fig. 1A). This action was defined as one trial, and one session was composed of 100 trials or a maximum of 45 min. After three sessions in this condition, the mice were trained to choose any one of the illuminated spots and receive a reward pellet with a sound. This condition comprised 100 trials or a maximum of 45 min per session and was conducted for 3–11 sessions. One mouse in the LKM + Arg-treated group died during pretest shaping.

### Behavioural sequencing task

The behavioural sequencing task began after acclimation (LKM + Arg: *n* = 11, control: *n* = 12). Briefly, the mice were required to discriminate between two rewarded and two never-rewarded spots and to shuttle between two rewarded spots positioned diagonally (Fig. 1B). Rewarded spots included one active and one inactive spot. If the mice chose the active spot, they received a reward pellet with a sound, and the assignation of active and inactive spots became alternatively changed as the trial proceeded. If the mice chose the inactive or never-rewarded spots, the house lamp was turned off for 5 s. This action was repeated until the mice chose the active spot, which was defined as an identical trial. The assignation of rewarded and never-rewarded spots was counterbalanced between and within groups, and the active spot in the first trial of each session was assigned to the highest spot (Spot 2 or Spot 3, Fig. 1B). The behavioural sequencing task comprised 150 trials or a maximum of 60 min per session and was conducted for 10 sessions.

### Serial reversal task

A serial reversal task was conducted after the behavioural sequencing task. Diagonal spatial patterns of rewarded and never-rewarded spots changed reversely every 5 or 10 sessions (Rev. 1–Rev. 4, Fig. 1C). The mice were required to shift from the previously acquired shuttling behaviour on one diagonal spatial pattern to the other diagonal spatial pattern. One mouse in the LKM + Arg-treated group did not take part in the second reversal phase (Rev. 2) because of poor physical condition. Five mice of the LKM + Arg-treated group and six mice of the control group were brain sampled at the beginning of the third reversal phase (Rev. 3). Although the number of c-Fos-positive cells in the prelimbic cortex for three mice per group was examined, no significant group differences were found (data not shown). Thus, the number of individuals included in the data analysis is as follows: (KM + Arg: *n* = 11, control: *n* = 12 at Rev. 1; LKM + Arg: *n* = 10, control: *n* = 12 at Rev. 2; and LKM + Arg: *n* = 5, control: *n* = 6 at Rev. 3 and Rev. 4.

### Behavioural assessment index

Learning performance during the test was assessed by the following behavioural assessment indices.

1. First selection discrimination error rate: percentage of never-rewarded spots (note that these are different from inactive spots) chosen at the first selection in a trial.
2. The rates of three movement patterns immediately after the correct choice: diagonal movement (in the first selection in a trial, diagonal movement from the correct spot in the immediately preceding trial to the following correct spot), adjacent movement (in the first selection in a trial, move to either of two adjacent spots from the correct spot in the immediately preceding trial), and re-entry (in the first selection in a trial, the identical spot as the correct spot in the immediately preceding trial).
3. Diagonal correct and error: diagonal movement between the two rewarded spots on the correct diagonal line in the first selections of each trial was considered a ‘diagonal correct’ selection. The diagonal movement between the two never-rewarded spots on the incorrect diagonal line in the first selection of each trial was considered a ‘diagonal error’ selection (Fig. 1C). Each selection was cumulatively calculated, and each value was subtracted for each trial as follows:
4. Diagonal behavioural index: (cumulative diagonal corrects − cumulative diagonal errors) within the first selection of the first 150 trials of each reversal phase. Thus, if the value increases negatively, the frequency of diagonal movement on the incorrect diagonal line is higher than that on the correct diagonal line. If the value changes to increase positively, the frequency of diagonal movement on the correct diagonal line is higher than that on the incorrect diagonal line.
5. Total trials to response contingency: in each reversal, the number of trials in which the minimum diagonal behavioural index (cumulative diagonal corrects − cumulative diagonal errors) was detected and then that value was no longer detected. This index was used to analyse adaptation speed to the response contingency.

### Statistical analysis

Daily data collected during the experiment were extracted as a text file and analysed using Excel (Microsoft). Data are indicated as mean ± standard error of the mean (SEM). For behavioural data analysis, we used a two-way repeated measures ANOVA with the Bonferroni *post hoc* test for multiple comparisons. *P* < 0.05 was considered statistically significant. All statistical analyses were performed using GraphPad Prism 9.

## Results

### Learning progressions in the behavioural sequencing task

**Figure 2:**
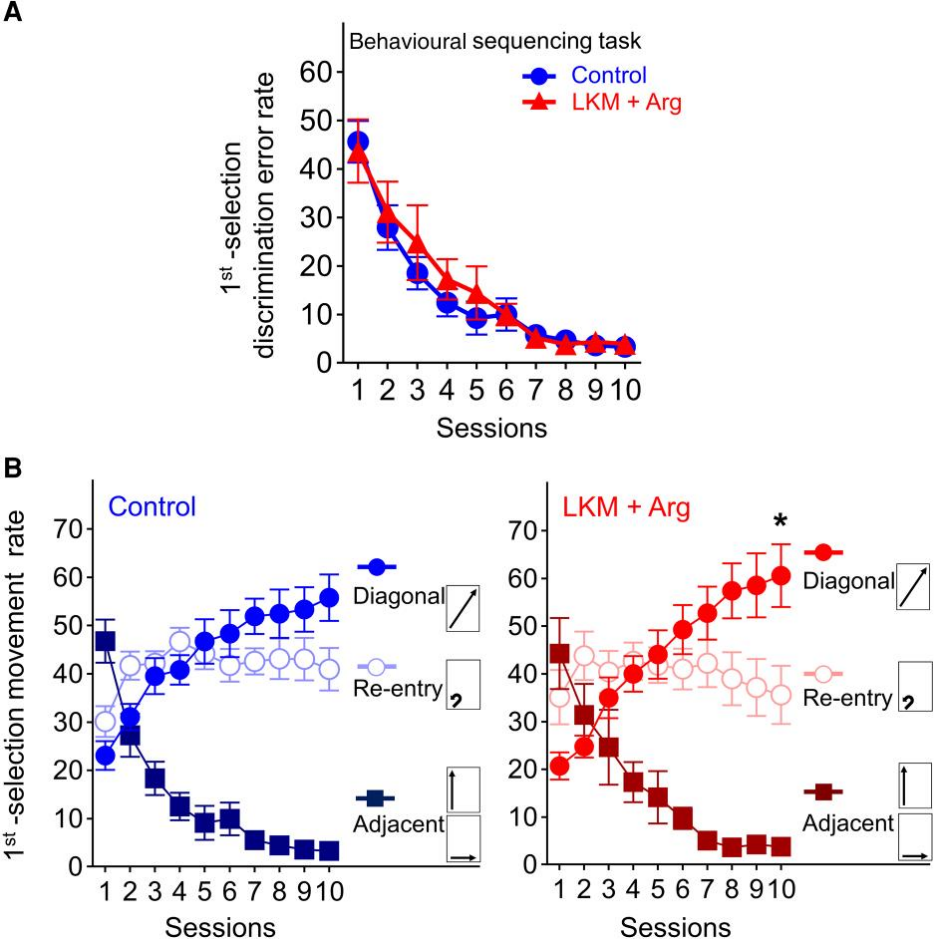
**Learning performance of the behavioural sequencing task.** (**A**) First selection discrimination error rate, defined as the number of choices of the two never-rewarded spots. The chance value is 50%. The error rate in both groups gradually decreased, indicating successful learning of the positions of never-rewarded spots. Each data point and error bar represent the mean and SEM of each group (LKM + Arg: *n* = 11, control: *n* = 12). (**B**) Rates of three movement patterns classified as follows: (i) diagonal movement; (ii) adjacent movement (to either of the two adjacent spots); and (iii) re-entry to the identical spot; accordingly, chance values were calculated as 25, 50 and 25%, respectively. *P < 0.05, significantly higher than re-entry and adjacent movement rate by two-way repeated measures ANOVA with Bonferroni correction. At Session 10, the diagonal movement rate was significantly higher than the re-entry and adjacent movement rate in the LKM + Arg-treated group (*P < 0.05). Each data point and error bar represent the mean and SEM of each group (LKM + Arg: *n* = 11, control: *n* = 12).

The first selection discrimination error rate on the first session was approximately equal to chance (LKM + Arg-treated group: 43.7% ± 6.5%, control group: 45.7% ± 4.3%), decreasing to <5% at the final session in both groups (LKM + Arg-treated group: 3.9% ± 1.2%, control group: 3.3% ± 0.7%; Fig. 2A). Both groups showed a concurrent increase to above the chance level in the diagonal movement selection rate (LKM + Arg-treated group: 20.7% ± 2.9–60.6% ± 6.6%, control group; 23.1% ± 3.0–55.8% ± 4.8%), and a remarkable decrease below the chance level in the adjacent movement selection rate (LKM + Arg-treated group: 44.3% ± 7.5–3.8% ± 1.2%, control group: 46.8% ± 4.5–3.3% ± 0.7%). Although the re-entry rate in control group tended to increase (30.1% ± 3.2–40.9% ± 4.4%), it did not change in the LKM+ Arg-treated group (32.5% ± 5.8–33.7% ± 6.1%). The diagonal movement significantly increased compared to other movement choices at Session 10 in the LKM + Arg-treated group (two-way repeated measures ANOVA with the Bonferroni *post hoc* test for multiple comparisons, session, *F*(9) < 0.0001, *P* > 0.9999, movement, *F*(2) = 18.74, *P* < 0.0001, session × movement, *F*(9,2) = 15.29, *P* < 0.0001, *post hoc*, Session 10, diagonal versus adjacent *P* < 0.0001, diagonal versus re-entry *P* = 0.0235, Fig. 2B). These results indicate that both groups learned the shuttling sequence.

### Discrimination performance in serial reversal task

**Figure 3:**
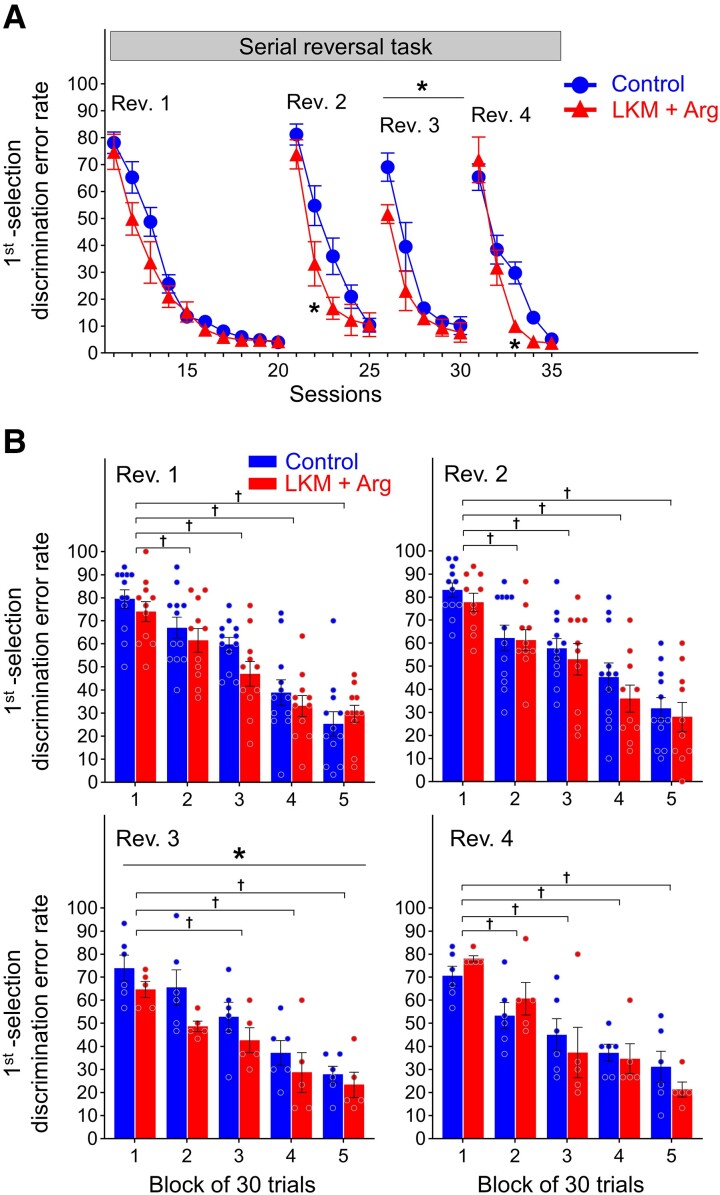
**Learning performance of serial reversal task.** (**A**) First selection discrimination error rate, defined as the number of choices of never-rewarded spots per session. *P < 0.05 represents significant differences between groups by two-way repeated measures ANOVA with Bonferroni correction. The first reversal phase, second reversal phase, third reversal phase and fourth reversal phase are presented as Rev. 1, Rev. 2, Rev. 3 and Rev. 4, respectively. Each data point and error bar represent the mean and SEM of each group (LKM + Arg: *n* = 11, control: *n* = 12 at Rev. 1; LKM + Arg: *n* = 10, control: *n* = 12 at Rev. 2; and LKM + Arg: *n* = 5, control: *n* = 6 at Rev. 3 and Rev. 4). (**B**) First selection discrimination error rates for every 30-trial block in the first 150 trials per reversal phase. *P < 0.05 represents significantly lower than control group over 150 trials by two-way repeated measures ANOVA. †*P* < 0.05 represents significantly lower than the first block by two-way repeated measures ANOVA with Bonferroni correction. The error rate decreased in both groups with each trial block (†*P* < 0.05). Especially at Rev. 3, the error rate was significantly lower in LKM + Arg-treated group than that in control group within the first 150 trials (*P < 0.05). Each bar and error bar represent the mean and SEM of each group, and each circle represents individual mouse data (LKM + Arg: *n* = 11, control: *n* = 12 at Rev. 1; LKM + Arg: *n* = 10, control: *n* = 12 at Rev. 2; and LKM + Arg: *n* = 5, control: *n* = 6 at Rev. 3 and Rev. 4).

The first selection discrimination error rates in all sessions in the serial reversal task are shown in Fig. 3A and Supplementary Table 1. At the first session of the first reversal phase (Session 11), the first selection discrimination error rates were above chance (50%) and were increased remarkably in both groups (LKM + Arg-treated group: 74.7% ± 6.5%, control group: 78.1% ± 4.0%) compared with the last session of the behavioural sequencing task (Session 10, Fig. 2A). Those rates decreased to <5% in both groups (LKM + Arg-treated group: 4.4% ± 1.1%, control group: 4.0% ± 0.8%) at the last session of Rev. 1 (Session 20).

In the second reversal phase (Rev. 2), those rates on the first session (Session 21) remarkably increased in both groups (LKM + Arg-treated group: 73.8% ± 5.4%, control group: 81.2% ± 3.9%) compared to the last session of Rev. 1. In this phase, on the second session in Rev. 2 (Session 22), those rates in the LKM + Arg-treated group were below the chance level (33.1% ± 8.3%), while that in the control group was still above chance (54.8% ± 7.4%). The error rate in the second session of Rev. 2 (Session 22) in the LKM + Arg-treated group was significantly lower than that in the control group (two-way repeated measures ANOVA with the Bonferroni *post hoc* test for multiple comparisons, session, *F*(4) = 109.6, *P* < 0.0001, group, *F*(1) = 3.176, *P* = 0.0899, session × group, *F*(4,1) = 3.077, *P* = 0.0207, *post hoc*, Session 22, *P* = 0.0354, Fig. 3A).

In the third reversal phase (Rev. 3), the first selection discrimination error rates on the first session (Session 26) were greater in both groups than the last session of Rev. 2. However, on the first session of Rev. 3, those rates were lower in both groups than the first session of Rev. 1 and Rev. 2. The rate in the LKM + Arg-treated group was almost equal to chance (51.6% ± 3.5%) while that in the control group was higher than chance (69.0% ± 5.3%). Throughout all sessions in Rev. 3, these rates in the LKM + Arg-treated group were significantly lower than that in the control group (two-way ANOVA with repeated measures, session, *F*(4) = 42.09, *P* < 0.0001, group, *F*(1) = 6.802, *P* = 0.0284, session × group, *F*(4,1) = 1.358, *P* = 0.2677, Fig. 3A).

In the fourth reversal phase (Rev. 4), the first selection discrimination error rates on the first session (Session 31) were greater in both groups than the last session of Rev. 3 (LKM + Arg-treated group: 71.7% ± 8.5%, control group: 65.3% ± 5.0%). The rate in the third session (Session 33) of Rev. 4 in the LKM + Arg-treated group was significantly lower than that in the control group (two-way repeated measures ANOVA with the Bonferroni *post hoc* test for multiple comparisons, session, *F*(4) = 127.1, *P* < 0.0001, group, *F*(1) = 1.735, *P* = 0.2203, session × group, *F*(4,1) = 4.415, *P* = 0.0052, *post hoc*, Session 33, *P* = 0.0127, Fig. 3A).

We next analysed the change in first selection discrimination error rate at the beginning of each reversal phase. Figure 3B and Supplementary Table 2 show the rates every 30 trials in the first 150 trials of each reversal phase. In Rev. 1, these rates gradually decreased in both groups to the fifth block. In the third block, those mean rate in the control group exceeded the chance level (50%) in the control group (59.7% ± 3.1%) but was already below the chance level in the LKM + Arg-treated group (47.0% ± 5.3%). In Rev. 2, those rates gradually decreased in both groups to the fifth block and below chance level from the fourth block (LKM + Arg-treated group: 36.0% ± 5.9%, control group: 45.3% ± 6.1%). In Rev. 3, the mean rate in the LKM + Arg-treated group was below chance from the second block while that in the control group was below chance from the fourth block. Through the blocks in Rev. 3, those rates in the LKM + Arg-treated group were significantly lower than that in the control group (two-way ANOVA with repeated measures, block, *F*(4) = 19.13, *P* < 0.0001, group, *F*(1) = 6.879, *P* = 0.0277, block × group, *F*(4,1) = 0.3082, *P* = 0.8706, Fig. 3B). In Rev. 4, those rates gradually decreased in both groups to the fifth block. The error rate in both groups was below chance from the third block onwards.

### Adaptation speed to response contingency in the serial reversal task

**Figure 4:**
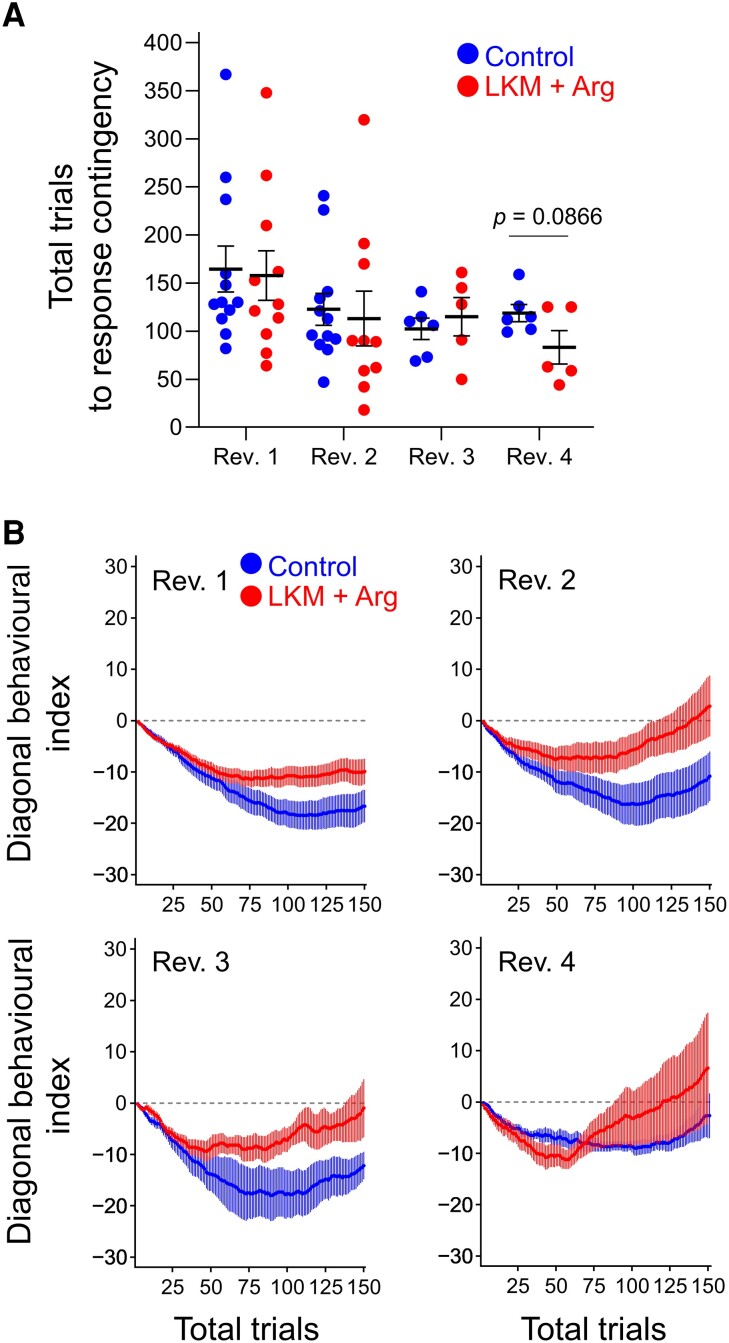
**Behavioural indices of responses to reversed contingency.** (**A**) Total trials to response contingency in each reversal phase (control: left side, LKM + Arg: right side). Each value represents the total number of trials where the minimum of diagonal behavioural indices (cumulative diagonal corrects − cumulative diagonal errors) was last detected in each reversal phase. The first reversal phase, second reversal phase, third reversal phase and fourth reversal phase are presented as Rev. 1, Rev. 2, Rev. 3 and Rev. 4, respectively. The value gradually decreased over repeating reversal phases in both groups. Each horizontal line and error bar represent the mean and SEM of each group, and each circle represents individual mouse data (LKM + Arg: *n* = 11, control: *n* = 12 at Rev. 1; LKM + Arg: *n* = 10, control: *n* = 12 at Rev. 2; and LKM + Arg: *n* = 5, control: *n* = 6 at Rev. 3 and Rev. 4). (**B**) Curves of diagonal behavioural indices within the first 150 trials in each reversal phase. Horizontal dotted lines represent zero value. Each circle and error bar represent the mean and SEM of each group (LKM + Arg: *n* = 11, control: *n* = 12 at Rev. 1; LKM + Arg: *n* = 10, control: *n* = 12 at Rev. 2; and LKM + Arg: *n* = 5, control: *n* = 6 at Rev. 3 and Rev. 4).

Next, we analysed the adaptation speed to response contingency by subtracting the cumulative number of diagonal errors from the cumulative number of diagonal corrects from the first trial at the beginning of each reversal phase. Figure 4A and Supplementary Table 3 (upper) show the total trials to response contingency from the beginning of each reversal phase. In Rev. 1, the total trials to response contingency in both groups were >150 trials (LKM + Arg-treated group: 157.8 ± 25.8 trials, control group: 164.5 ± 23.9 trials). However, this number gradually decreased to the Rev.4 (LKM + Arg-treated group: 83.2 ± 17.4 trials, control group: 118.8 ± 8.9 trials); the timing of the shift to reversed contingency is gradually faster with repeating reversals. Interestingly, the LKM + Arg-treated group tended to shift to reversed contingency faster than the control group in Rev. 4 (Student’s *t*-test, *t* = 1.923, *P* = 0.0866, Fig. 4A).

The diagonal behavioural index, which is shown in Fig. 4B, decreased and then increased in all reversal phases in both groups. In the first reversal phase (Rev. 1), no remarkable increase of the value of diagonal behavioural index was observed by the 150th trial in any group. However, compared to the control group, the LKM + Arg-treated group was less likely to perform diagonal error movements. The minimum diagonal behavioural indices in the LKM + Arg-treated and control groups, which are shown in Supplementary Table 3 (under), were −14.7 ± 2.6 and −20.6 ± 2.9, respectively. In the second reversal phase (Rev. 2) and subsequent ones, a gradual increase in the diagonal behavioural index was observed within the 150th trial in both groups. In Rev. 2, the minimum values of the index in the LKM + Arg-treated group and the control group were −12.5 ± 3.0 and −19.3 ± 4.4, respectively; in Rev. 3, −12.2 ± 1.9 and −21.0 ± 4.7; and in Rev. 4, −13.0 ± 1.8 and −9.8 ± 1.3.

These results indicate that both groups showed behavioural flexibility with repeating reversals. Particularly, the LKM + Arg-treated group suppressed more their diagonal error movement, which was the previously correct one, to shift to a diagonal correct movement than the control group, and the LKM + Arg-treated group was faster than the control group to shift from diagonal error movement to diagonal correct movement.

## Discussion

The present study showed the effect of long-term treatment with LKM + Arg on cognitive flexibility in middle-aged mice. The cognitive flexibility task demands mice to respond to reversed contingency while maintaining a shuttling behaviour in a serial reversal task. We have previously shown that mice show a progressive adaptation to contingency with repeating reversal changes, indicating that mice acquire an adaptation to the ‘reversal rule’ itself.^12-14,25^ LKM + Arg-treated mice showed an early onset of shift to reversal contingency and thus improved cognitive flexibility. A previous study assessing spontaneous cognitive declines in C57BL/6 mice has reported that the mice show the deteriorated spatial learning and memory measured by the Morris water maze test and recognition memory measured by the novel object recognition test from 6 months of age, worsening at 12 months of age.^26^ Additionally, compared to our previous cognitive flexibility task data from young mice (9–23 weeks old), in the present study, we found that the discrimination error rates on the first session of the serial reversal task in control mice in this study were >20% higher than those in young mice.^13^ These findings indicate that 13 months old is the age when cognitive decline is significant, including cognitive flexibility, and that the probiotic treatment reduces the decline in cognitive flexibility. Our findings could serve to promote preventive interventions focusing on cognitive flexibility, which is impaired in aging and early stages of dementia.

Few studies have investigated the impact of probiotics on higher cognitive function in animals because of the limited cognitive behavioural tasks available to this end. We reported that acquired experience is stored in the brain as a schema, and that schema processing can be updated to respond quickly to a different task.^27^ In the LKM + Arg-treated group, the performance of choosing never-rewarded spots, which were previously rewarded spots, and the diagonal error movement were suppressed at the beginning of each reversal phase. Thus, LKM + Arg may induce a rapid updating of the schema to reversed contingency. In the behavioural sequence task, equivalent to spatial learning, there was no remarkable difference in shuttling behaviour on the diagonal correct line between the LKM + Arg-treated group and control group. In other words, treatment with LKM + Arg may affect more cognitive flexibility than learning and memory of behavioural sequencing. To clarify the sensitivity of the treatment, future experiments are required to validate difference of effect of treatment with LKM + Arg on cognitive flexibility by comparing different treatment durations and doses.

We previously reported that long-term treatment with LKM + Arg affected the regulation of metabolites in the prefrontal cortex^24^; thus, the probiotic treatment can impact the cognitive function in mice in the present study. However, it is important to note that the results in this study can also be interpreted as an improvement of stubbornness-like behaviour, which comprises psychological problems. Further validation of the effect of the treatment on both aspects of cognitive and psychological changes is needed by refining experimental protocols to clearly indicate the aspects influenced.

Polyamine treatments can reduce age-associated pathology and extend lifespan in mice^28,29^; thus, polyamines are considered important factors for improving quality of life, delaying cognitive decline during aging and preventing dementia. A previous study reports that treatment with LKM + Arg induces two independent metabolic pathways to produce polyamines.^30^ The first pathway is that environmental acidification triggers the Arg-dependent acid tolerance system of *Escherichia coli*, and agmatine is produced by the system.^30^ The second pathway is that energy production system by *Enterococcus faecalis* is induced via agmatine, resulting in synthesis of putrescine.^30^ Moreover, putrescine is converted to spermidine and transferred into the bloodstream, and spermidine improves vascular endothelial function.^31^ In the present study, we found that treatment with LKM + Arg had positive effects on cognitive function in middle-aged mice. Previous studies assessing changes in intestinal microbiota with LKM512 treatment have reported that relative abundance of the Firmicutes species decreases in mice,^23^ and that the Firmicutes/*Bacteroides* ratio decreases in humans.^32^ Therefore, the same effect of changes on intestinal microbiota as that in the previous studies can be observed in mice with probiotic treatment. In the present study, changes in intestinal microbiota, including polyamines, may be involved in cognitive flexibility. However, the link between intestinal microbiota-derived polyamines and cognitive flexibility remains unclear. It is possible that the polyamines produced in the intestine could have produced metabolites that influenced the improvement of cognitive flexibility as there are no reports of polyamines directly permeating across the blood–brain barrier. Further research is warranted to elucidate the mechanism underlying microbiota-derived polyamine–gut–brain signalling pathways.

In conclusion, long-term treatment with LKM + Arg improves cognitive flexibility in middle-aged mice. These findings provide insight for future application to intervention methods as function foods in cognitive decline of aging and early stages of dementia.

## Supplementary Material

fcad311_Supplementary_Data

## Data Availability

The data that support the findings of this study are available from the corresponding author, upon reasonable request. The data of serial reversal task can be found in the Supplementary material.
