# Supplementary material for: Probiotic treatment with Bifidobacterium animalis subsp. lactis LKM512 + arginine improves cognitive flexibility in middle-aged mice
Source: Brain Commun. 2023 Nov 13;5(6):fcad311. doi: 10.1093/braincomms/fcad311 (PMC10667025; doi:10.1093/braincomms/fcad311)
Supplement: fcad311_Supplementary_Data [file fcad311_supplementary_data.pdf]

Supplementary Table 1. The first selection discrimination error rate in each session of each reversal phase. Mean ± SEM.

**Rev. 1**					
Session	11	12	13	14	15
Control	78.1 ± 4.0	65.2 ± 5.9	48.7 ± 5.4	25.7 ± 3.4	13.5 ± 1.5
LKM + Arg	74.7 ± 6.5	49.9 ± 6.1	33.6 ± 7.7	21.0 ± 4.0	15.3 ± 3.7
Session	16	17	18	19	20
Control	11.7 ± 2.0	8.1 ± 1.5	5.9 ± 1.0	4.9 ± 0.6	4.0 ± 0.8
LKM + Arg	8.7 ± 2.4	6.0 ± 1.1	4.8 ± 0.9	4.8 ± 0.9	4.4 ± 1.1
**Rev. 2**					
Session	21	22	23	24	25
Control	81.2 ± 3.9	54.8 ± 7.4	36.0 ± 6.8	21.0 ± 4.3	10.4 ± 2.5
LKM + Arg	73.8 ± 5.4	33.1 ± 8.3	16.6 ± 4.1	12.3 ± 5.8	10.5 ± 4.4
**Rev. 3**					
Session	26	27	28	29	30
Control	69.0 ± 5.3	39.5 ± 9.0	16.6 ± 1.7	11.7 ± 1.2	10.2 ± 3.3
LKM + Arg	51.6 ± 3.5	23.1 ± 7.3	12.9 ± 2.2	9.5 ± 3.2	7.7 ± 3.7
**Rev. 4**					
Session	31	32	33	34	35
Control	65.3 ± 5.0	38.4 ± 5.3	29.7 ± 4.1	13.1 ± 2.1	5.1 ± 1.0
LKM + Arg	71.7 ± 8.5	31.7 ± 6.6	10.0 ± 1.9	4.3 ± 0.8	3.7 ± 0.9

Supplementary Table 2. The first selection discrimination error rate every 30 trials within the first 150 trials of each reversal phases. Mean ± SEM.

Rev. 1					
Block of 30 trials	1	2	3	4	5
Control	79.4 ± 4.0	66.9 ± 4.6	59.7 ± 3.1	38.9 ± 5.5	25.3 ± 5.3
LKM + Arg	73.9 ± 4.4	61.5 ± 5.2	47.0 ± 5.3	33.0 ± 4.5	29.7 ± 3.7
**Rev. 2**					
Block of 30 trials	1	2	3	4	5
Control	83.1 ± 3.1	62.2 ± 5.6	57.8 ± 4.3	45.3 ± 6.1	31.7 ± 4.7
LKM + Arg	77.7 ± 3.9	61.3 ± 4.5	53.0 ± 6.8	36.0 ± 5.9	28.0 ± 6.4
**Rev. 3**					
Block of 30 trials	1	2	3	4	5
Control	73.9 ± 5.7	65.6 ± 7.6	52.8 ± 6.3	37.2 ± 5.3	27.8 ± 3.6
LKM + Arg	64.7 ± 3.4	48.7 ± 2.3	42.7 ± 5.4	28.7 ± 8.7	23.3 ± 5.5
**Rev. 4**					
Block of 30 trials	1	2	3	4	5
Control	70.6 ± 4.2	53.3 ± 5.7	45.0 ± 7.0	37.2 ± 3.7	31.1 ± 6.8
LKM + Arg	78.0 ± 1.3	60.7 ± 7.0	37.3 ± 10.9	34.7 ± 6.5	21.3 ± 3.3

Supplementary Table 3. Total trials to response contingency (upper) and minimum diagonal behavioral index (under) in each reversal phase. Mean ± SEM.

Total trials to response contingency				
	Rev. 1	Rev. 2	Rev. 3	Rev. 4
Control	164.5 ± 23.9	122.8 ± 16.6	102.3 ± 11.1	118.8 ± 8.9
LKM + Arg	157.8 ± 25.8	113.1 ± 28.6	115.0 ± 20.0	83.2 ± 17.4

Minimum diagonal behavioral index				
	Rev. 1	Rev. 2	Rev. 3	Rev. 4
Control	−20.6 ± 2.9	−19.3 ± 4.4	−21.0 ± 4.7	−9.8 ± 1.3
LKM + Arg	−14.7 ± 2.6	−12.5 ± 3.0	−12.2 ± 1.9	−13.0 ± 1.8
